# Association of maternal smoking, breastfeeding, and multiple birth with irritable bowel syndrome in older adults: a UK Biobank cohort study

**DOI:** 10.1093/gastro/goaf042

**Published:** 2025-06-11

**Authors:** Xinyang Liu, Ruilang Lin, Xinyue Li, Mengjiang He, Yanbo Liu, Jianwei Hu, Weifeng Chen, Quanlin Li, Yongfu Yu, Pinghong Zhou

**Affiliations:** Endoscopy Center and Endoscopy Research Institute, Zhongshan Hospital, Fudan University, Shanghai, P. R. China; Shanghai Collaborative Innovation Center of Endoscopy, Shanghai, P. R. China; Department of Biostatistics, School of Public Health, and The Key Laboratory of Public Health Safety of Ministry of Education, Fudan University, Shanghai, P. R. China; NHC Key Laboratory of Health Technology Assessment, Fudan University, Shanghai, P. R. China; Endoscopy Center and Endoscopy Research Institute, Zhongshan Hospital, Fudan University, Shanghai, P. R. China; Shanghai Collaborative Innovation Center of Endoscopy, Shanghai, P. R. China; Endoscopy Center and Endoscopy Research Institute, Zhongshan Hospital, Fudan University, Shanghai, P. R. China; Shanghai Collaborative Innovation Center of Endoscopy, Shanghai, P. R. China; Endoscopy Center and Endoscopy Research Institute, Zhongshan Hospital, Fudan University, Shanghai, P. R. China; Shanghai Collaborative Innovation Center of Endoscopy, Shanghai, P. R. China; Endoscopy Center and Endoscopy Research Institute, Zhongshan Hospital, Fudan University, Shanghai, P. R. China; Shanghai Collaborative Innovation Center of Endoscopy, Shanghai, P. R. China; Endoscopy Center and Endoscopy Research Institute, Zhongshan Hospital, Fudan University, Shanghai, P. R. China; Shanghai Collaborative Innovation Center of Endoscopy, Shanghai, P. R. China; Endoscopy Center and Endoscopy Research Institute, Zhongshan Hospital, Fudan University, Shanghai, P. R. China; Shanghai Collaborative Innovation Center of Endoscopy, Shanghai, P. R. China; Department of Biostatistics, School of Public Health, and The Key Laboratory of Public Health Safety of Ministry of Education, Fudan University, Shanghai, P. R. China; NHC Key Laboratory of Health Technology Assessment, Fudan University, Shanghai, P. R. China; Endoscopy Center and Endoscopy Research Institute, Zhongshan Hospital, Fudan University, Shanghai, P. R. China; Shanghai Collaborative Innovation Center of Endoscopy, Shanghai, P. R. China

**Keywords:** irritable bowel syndrome, perinatal early-life factors, UK Biobank, Townsend Deprivation Index

## Abstract

**Background:**

We aimed to investigate associations between three perinatal early-life factors and the risk of irritable bowel syndrome (IBS) in middle-aged and elderly people by using data from UK Biobank.

**Methods:**

This is a population-based cohort study. Participants who had available data on early-life factors—namely maternal smoking around birth, being breastfed as a baby, and being one of a multiple birth and without IBS at the time of recruitment in UK Biobank—were included.

**Results:**

Among a total of 334,586 subjects, 93,908 (28.07%) were exposed to maternal smoking around birth, 243,778 (72.86%) were breastfed as a baby, and 7,551 (2.26%) were part of a multiple birth. During a median follow-up of 13.58 years, 7,254 participants developed IBS, at a median age of 63 years. The hazard ratios of IBS were 1.22 [95% confidence interval (CI), 1.16–1.28, *P *<* *0.001], 0.92 (95% CI, 0.87–0.97, *P *=* *0.002), and 1.22 (95% CI, 1.06–1.40, *P *=* *0.006) for maternal smoking, breastfeeding, and multiple birth, respectively. The joint effect of any two of these three factors was related to added influence instead of interaction between them. The effect of maternal smoking on IBS was modified by age, while the modifiers of the effect of being breastfed as a baby on IBS were the age and sex of the offspring.

**Conclusions:**

Participants exposed to maternal smoking around birth and being one of a multiple birth had a higher risk of IBS in middle-aged and elderly stages, while being breastfed as a baby had a protective effect against IBS. Future efforts should be made to validate the results.

## Introduction

Irritable bowel syndrome (IBS) is a functional gastrointestinal disorder causing recurrent abdominal pain and alterations in bowel habits, which affects 5%–10% of the general population [1]. It is one of the major reasons for gastroenterology consultations in primary and secondary care, with a substantial impact on quality of life and social functioning [2, 3]. The pathophysiology of IBS is only partially understood, although previous acute enteric infection [4] and psychological factors [5, 6] have been established as risk factors. Moreover, many other social and environmental factors are also linked to IBS and its severity [1]. However, the impact of perinatal exposures on offspring’s IBS risk in adulthood has received relatively limited attention.

Both maternal smoking and breastfeeding could influence the offspring’s gut health through nutritional and microbiome-mediated pathways [7, 8]. Maternal smoking was also found to be associated with increased risk of psychological disorders in the offspring [9]. Interestingly, recent studies also uncovered a potential risk of psychiatric disorders as a long-term effect of being in a multiple birth [10, 11]. These aforementioned conditions, such as psychological comorbidity and gut health, are closely related to IBS. Nonetheless, whether these perinatal early-life factors are associated with the risk of IBS in offspring remains to be answered. Previous studies have suggested interactions between maternal smoking and breastfeeding in the context of offspring psychological disorders [12], hypertension [13], and eczema [14]. Moreover, as these early-life factors are associated with each other to some extent as intervening variables, it is also of interest to explore the joint effects in addition to their respective independent effects.

In this study, using data from UK Biobank, we aimed to examine the associations between three perinatal factors, including maternal smoking around birth, being breastfed as a baby, and singleton or multiple births, and the risk of IBS in offspring. Furthermore, we sought to investigate the combined effects of these three factors and explore potential effect modification by age, sex, and offspring smoking status.

## Materials and methods

### Study population

UK Biobank is a large, population-based, multicenter prospective cohort study that recruited >500,000 middle-aged and elderly participants from 22 assessment centres across England, Wales, and Scotland between 2006 and 2010 [15, 16]. Data on lifestyles, physical measures, biological samples, and medical conditions are available on the website of UK Biobank (www.ukbiobank.ac.uk). The UK Biobank study was approved by North West Multicenter Research Ethical Committee and conformed to the principles outlined in the Declaration of Helsinki. Written informed content was obtained from all participants.

**Figure 1.**
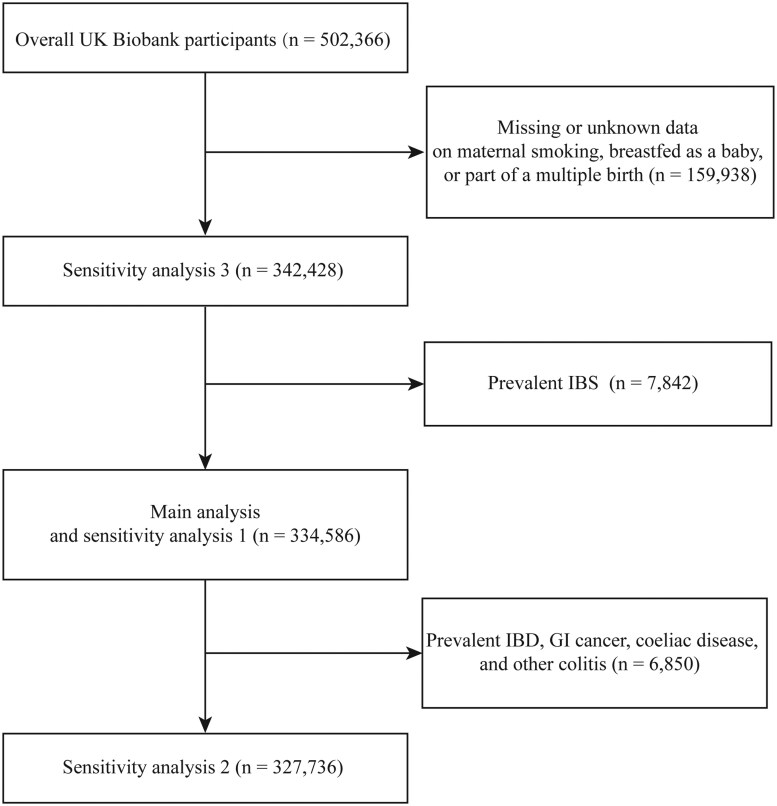
Flow chart of participant selection. IBD = inflammatory bowel disease, GI = gastrointestinal.

The current study included participants with available data on early-life factors— namely maternal smoking around birth, being breastfed as a baby, and being one of a multiple birth—and without IBS at the time of recruitment. These criteria yielded a total of 334,586 participants in the main analysis. We compared the primary characteristics between participants who were included in the primary analysis and those who did not report prevalent IBS and were excluded due to missing or unknown data on any early-life factor (Supplementary Table 1). A flowchart of the participant selection is illustrated in Figure 1.

### Assessment of exposures

Exposures of interest included maternal smoking (yes/no), breastfeeding (yes/no), and parity (singleton/multiple). The relevant information can be found in the Field ID 1787, 1677, and 1777 under ‘Early life factors’ (Field ID 100033) category.

### Ascertainment of outcomes

The primary outcome of interest was incident IBS. Data on incident IBS were extracted from the hospital inpatient records of each participant according to an International Classification of Diseases 10th revision code of K58.0 or K58.9 in the Field ID 41270. At the time of analysis, death data were available up to 30 November 2022. Hospital-admission data were available until 31 August 2022 for Scotland, 31 May 2022 for Wales, and 31 October 2022 for England. Therefore, for the event outcomes, the follow-up was censored at these dates unless a relevant hospitalization or death from any cause occurred that preceded these points.

### Covariates and other variables included in the study

Covariates included age at recruitment, sex, ethnicity, education level, Townsend Deprivation Index, body mass index (BMI), smoking status, and alcohol-drinking status. Detailed information on covariates are listed in Supplementary Table 2. Prevalent cases of IBS, inflammatory bowel disease (IBD), gastrointestinal cancer, coeliac disease, and other colitis at recruitment were ascertained from ‘Non-cancer illness code, self-reported’ and ‘Cancer illness code, self-reported’ (Field ID 20002 and 20001).

### Statistical analysis

Baseline characteristics were compared by using the *t*-test or chi-squared test. Incident IBS was treated as a time-to-event variable, with time defined as the age at diagnosis of IBS or censored at the age of death or the age of loss to follow-up, whichever occurred first. Cox regression models were used to explore the associations between exposures and incident IBS, and the results are presented as hazard ratios (HRs) and 95% confidence intervals (CIs).

To elucidate the relationships between early-life factors and the risk of adult-onset IBS, we conducted both unadjusted and adjusted regression analyses. Potential confounders and mediators were identified by using a Directed Acyclic Graph (Supplementary Figure 1). Being one of a multiple birth was considered a potential confounder and we further adjusted for it in testing the associations of maternal smoking and breastfeeding with IBS, but not vice versa, as per the Directed Acyclic Graph. We also investigated the joint effect of any two of the three early-life factors in the adjusted analyses. Effect modification by certain modifiers was tested by using a likelihood ratio test, comparing models with and without the interaction terms with the exposure based on the adjusted Cox model. Different strata of sex, age, and smoking status were then taken as subgroups to show the associations between these exposures and IBS.

We performed a series of sensitivity analyses to examine the robustness of the results. In Sensitivity Analysis 1, potential mediators that were identified by using the Directed Acyclic Graph were further introduced into the adjusted model to test the potential influence on the results. In Sensitivity Analysis 2, to account for the influence of gastrointestinal organic conditions that might overlap with or be mistaken for IBS due to similar symptoms, we repeated the analysis after excluding participants with prevalent IBD, gastrointestinal cancer, coeliac disease, and other colitis. In Sensitivity Analysis 3, the analyses were repeated in the dataset before excluding 7,842 prevalent IBS cases. Logistic regression models were used to explore the associations between exposures and both prevalent and incident IBS in a case–control study format and the results are shown as odds ratios and 95% CIs.

Two-sided tests were used and a *P*-value of <0.05 was considered statistically significant. All statistical analyses were performed by using Stata 17.0 (StataCorp LLC, College Station, Texas, USA).

## Results

### Characteristics of included participants

Among 334,586 subjects in the main analysis, 93,908 (28.1%) were exposed to maternal smoking around birth, 243,778 (72.9%) were breastfed as a baby, and 7,551 (2.3%) were one of a multiple birth. Over a median follow-up of 13.58 years, 7,254 participants developed IBS. Compared with participants without IBS, those who developed IBS were characterized by older age at the time of recruitment, a higher likelihood of being female and of White ethnicity, and a Townsend Deprivation Index. The two groups differed in education level, smoking status, alcohol-drinking status, and BMI at the baseline time (Table 1).

**Table 1.** Baseline characteristics of included participants by incident IBS status

Variable	Incident IBS		*P*-value
	Yes (*n *=* *7,254)	No (*n *=* *327,332)	
Age at recruitment, years	56.2 ± 8.0	55.7 ± 8.2	<0.001
Sex			<0.001
Male	1,774 (24.5)	141,333 (43.2)	
Female	5,480 (75.6)	185,999 (56.8)	
Ethnicity			<0.001
White	6,532 (90.0)	290,788 (88.8)	
Non-White	699 (9.6)	35,616 (10.9)	
Unknown	23 (0.3)	928 (0.3)	
Maternal smoking around birth	2,360 (32.5)	91,548 (28.0)	<0.001
Breastfed as a baby	5,096 (70.3)	238,682 (72.9)	<0.001
Type of birth			0.004
Multiple	200 (2.8)	7,351 (2.2)	
Singleton	7,054 (97.2)	319,981 (97.8)	
Townsend Deprivation Index			<0.001
Quartile 1	1,688 (23.3)	81,954 (25.0)	
Quartile 2	1,694 (23.4)	81,759 (25.0)	
Quartile 3	1,812 (25.0)	81,716 (25.0)	
Quartile 4	2,052 (28.3)	81,485 (24.9)	
Unknown	8 (0.1)	418 (0.1)	
Education level			<0.001
Non-university	5,043 (69.5)	235,557 (72.0)	
University	723 (10.0)	41,008 (12.5)	
Unknown	1,488 (20.5)	50,767 (15.5)	
Smoking status			<0.001
Never	3,902 (53.8)	184,831 (56.5)	
Previous	2,548 (35.1)	108,754 (33.2)	
Current	781 (10.8)	32,730 (10.0)	
Unknown	23 (0.3)	1,017 (0.3)	
Alcohol drinking			<0.001
Never	422 (5.8)	15,421 (4.7)	
Previous	425 (5.9)	11,035 (3.4)	
Current	6,400 (88.2)	300,547 (91.8)	
Unknown	7 (0.1)	329 (0.1)	
BMI			<0.001
Underweight (<18.5 kg/m^2^)	51 (0.7)	1,691 (0.5)	
Normal (≥18.5 and <25 kg/m^2^)	2,364 (32.6)	109,501 (33.5)	
Overweight (≥25 and <30 kg/m^2^)	2,772 (38.2)	136,769 (41.8)	
Obese (≥30 kg/m^2^)	2,009 (27.7)	77,664 (23.7)	
Unknown	58 (0.8)	1,707 (0.5)	

Values are presented as mean ± standard deviation or numbers (percentage).

### Perinatal early-life factors and adult-onset IBS

Associations of perinatal early-life factors and the risk of IBS in offspring are listed in Table 2. In the unadjusted analysis, participants who were exposed to maternal smoking around birth had a 29% increased hazard of IBS (HR = 1.29, 95% CI, 1.23–1.36, *P *<* *0.001). The increase in hazard reduced significantly to 22% after adjusting for potential confounders including sex, age, ethnicity, education, Townsend Deprivation Index, and being one of a multiple birth, but remained statistically significant (HR = 1.22, 95% CI, 1.16–1.28, *P *<* *0.001). On the contrary, being breastfed as a baby showed a protective effect against adult-onset IBS in both unadjusted and adjusted analyses. It was associated with a 28% decrease in the hazard of developing IBS (HR = 0.72, 95% CI, 0.68–0.75, *P *<* *0.001) in the unadjusted analysis and the protective effect shrank to only 8% after adjusting for the aforementioned covariates (HR = 0.92, 95% CI, 0.87–0.97, *P *=* *0.002). Being one of a multiple birth was associated with a 24% increased hazard of developing IBS (HR = 1.24, 95% CI, 1.08–1.43, *P *=* *0.003) in the unadjusted analysis and the effect remained similar after adjusting for sex, age, and ethnicity (HR = 1.22, 95% CI, 1.06–1.40, *P *=* *0.006).

**Table 2.** Association between early-life factors and adult-onset IBS

Category of exposure	No. of outcomes	Unadjusted			Adjusted	
		Rate (per 100,000 person-years)	HR (95% CI)	*P*-value	HR (95% CI)	*P*-value
Maternal smoking around birth^a^						
No	4,894	29.38	Reference	–	Reference	–
Yes	2,360	36.55	1.29 (1.23, 1.36)	<0.001	1.22 (1.16, 1.28)	<0.001
Breastfed as a baby^a^						
No	2,158	35.61	Reference	–	Reference	–
Yes	5,096	29.88	0.72 (0.68, 0.75)	<0.001	0.92 (0.87, 0.97)	0.002
Type of birth^b^						
Singleton	7,054	31.22	Reference	–	Reference	–
Multiple	200	38.40	1.24 (1.08, 1.43)	0.003	1.22 (1.06, 1.40)	0.006

a Adjusted for sex, age, ethnicity, education level, Townsend Deprivation Index, and being one of a multiple birth.

b Adjusted for sex, age, and ethnicity.

### Joint effect of perinatal early-life factors on the risk of adult-onset IBS

We found that the offspring of mothers who smoked and did not breastfeed had a 31% higher risk of developing IBS (HR = 1.31, 95% CI, 1.21–1.41, *P *<* *0.001) compared with the offspring of mothers who breastfed and did not smoke (Table 3). Participants who were exposed to both maternal smoking and were one of a multiple birth had a 32% increased risk of IBS (HR = 1.32, 95% CI, 1.03–1.70, *P *=* *0.030) compared with those exposed to neither of those risk factors (Table 3). Moreover, participants who were not breastfed and were one of a multiple birth had a 38% elevated risk of developing IBS (HR = 1.38, 95% CI, 1.13–1.68, *P *=* *0.002) compared with those who were breastfed and of a singleton birth (Table 3). All the *P*-values for the interaction terms were not statistically significant, suggesting added influence instead of the interaction between the exposures.

**Table 3.** Joint effect of early-life factors on adult-onset IBS

Attributing effect	No. of outcomes	Rate (per 100,000 person-years)	HR (95% CI)	*P*-value
Maternal smoking and being breastfed as a baby				
Main effect				
No maternal smoking and with breastfeeding	3,622	28.42	Reference	–
Maternal smoking only	1,474	34.20	1.20 (1.13, 1.27)	<0.001
No breastfeeding only	1,272	32.50	1.05 (0.98, 1.12)	0.142
Joint effect				
Maternal smoking and no breastfeeding	886	41.28	1.31 (1.21, 1.41)	<0.001
*P*-value for interaction (maternal smoking × breastfeeding)				0.449
Maternal smoking and being one of a multiple birth				
Main effect				
No maternal smoking and no multiple birth	4,755	29.19	Reference	–
Maternal smoking only	2,299	36.45	1.22 (1.16, 1.29)	<0.001
Multiple birth only	139	37.45	1.24 (1.05, 1.47)	0.012
Joint effect				
Maternal smoking and multiple birth	61	40.75	1.32 (1.03, 1.70)	0.030
*P*-value for interaction (maternal smoking × one of a multiple birth)				0.259
Breastfed as a baby and one of a multiple birth				
Main effect				
Breastfeeding and no multiple birth	4,995	29.82	Reference	–
No breastfeeding only	2,059	35.24	1.08 (1.02, 1.14)	0.004
Multiple birth only	101	33.30	1.09 (0.89, 1.32)	0.413
Joint effect				
No breastfeeding and multiple birth	99	45.51	1.38 (1.13, 1.68)	0.002
*P*-value for interaction (breastfeeding × one of a multiple birth)				0.259

### Effect modification

Effect modification by sex and age (≥60 vs <60 years) on the associations between perinatal early-life factors and IBS was assessed by using a likelihood ratio test based on the adjusted model (Table 4). The smoking status of the participants was also examined for effect modification in the association between maternal smoking around birth and the outcome. Regarding the effect of maternal smoking around birth on offspring IBS, the interaction term with exposure was statistically significant for age (*P *=* *0.002), but not for sex (*P *=* *0.060) and smoking status of the offspring (*P *=* *0.527), indicating that the effects of maternal smoking around birth on offspring IBS were different between age subgroups, but consistent across different strata by sex and smoking status. The effect of maternal smoking on offspring IBS was higher when the offspring were younger (HR = 1.30, 95% CI, 1.22–1.39, *P *<* *0.001) and tended to decrease when they got older (HR = 1.11, 95% CI, 1.02–1.20, *P *=* *0.012). In terms of the effect of being breastfed as a baby on IBS, both the age and sex of the offspring were effect modifiers (*P *<* *0.001 and *P *=* *0.023, respectively). Breastfeeding demonstrated a protective effect on offspring IBS in male offspring (HR = 0.81, 95% CI, 0.72–0.89, *P *<* *0.001) but not in females (HR = 0.96, 95% CI, 0.90–1.02, *P *=* *0.147). Similarly, breastfeeding was associated with a 34% decreased risk of offspring IBS when the offspring were <60 years old (HR = 0.76, 95% CI, 0.71–0.81, *P *<* *0.001) and the effect faded when they were >60 years old (HR = 1.03, 95% CI, 0.94–1.13, *P *=* *0.496). For the effect of being one of a multiple birth on IBS, neither sex nor age showed effect modification.

**Table 4.** Effect modification on the associations between early-life factors and adult-onset IBS

Variable	No. of subjects	No. of outcomes	HR (95% CI)	*P*-value	*P* for interaction
Maternal smoking around birth					
Sex					0.060
Female	191,479	5,480	1.24 (1.17, 1.32)	<0.001	
Male	143,107	1,774	1.15 (1.04, 1.27)	0.006	
Age, years					0.002
<60	204,399	4,308	1.30 (1.22, 1.39)	<0.001	
≥60	130,187	2,946	1.11 (1.02, 1.20)	0.012	
Smoking status					0.527
Never	188,733	3,902	1.23 (1.15, 1.32)	<0.001	
Previous	111,302	2,548	1.22 (1.12, 1.32)	<0.001	
Current	33,511	781	1.07 (0.93, 1.24)	0.342	
Unknown	1,040	23	1.17 (0.45, 3.02)	0.746	
Breastfed as a baby					
Sex					0.023
Female	191,479	5,480	0.96 (0.90, 1.02)	0.147	
Male	143,107	1,774	0.81 (0.72, 0.89)	<0.001	
Age, years					<0.001
<60	204,399	4,308	0.76 (0.71, 0.81)	<0.001	
≥60	130,187	2,946	1.03 (0.94, 1.13)	0.496	
One of a multiple birth					
Sex					0.724
Female	191,479	5,480	1.23 (1.05, 1.45)	0.010	
Male	143,107	1,774	1.16 (0.87, 1.56)	0.313	
Age, years					0.816
<60	204,399	4,308	1.23 (1.03, 1.48)	0.021	
≥60	130,187	2,946	1.20 (0.96, 1.50)	0.115	

### Sensitivity analyses

The results of Sensitivity Analyses 1–3 are shown in Supplementary Tables 3–5 and were all consistent with the primary analysis, which indicated the robustness of the findings.

## Discussion

In this extensive population-based cohort study, we found that maternal smoking around birth and being one of a multiple birth were associated with a 22% increased risk of IBS in offspring during adulthood, while breastfeeding could reduce the hazard by 8% after adjusting for potential confounders. The joint effect of any two risk factors was also identified. The results were robust in sensitivity analyses.

Perinatal exposure to maternal smoking affects offspring development through both environmental and epigenetic pathways [17]. Previously, lots of efforts have been dedicated to clarifying the effect of maternal smoking on adverse outcomes during pregnancy, infancy, and early life [18], including low birthweight [19] and stillbirth [20]. Recently, associations between maternal smoking and long-term consequences such as cardiovascular diseases [21] and psychological disorders [22] have been studied. Concerning the gastrointestinal system, it was found to have an impact on the infant gut microbiota [9] and increase the risk of childhood IBD in offspring [8]. The advantages of breastfeeding for child health have been well established, including reduced mortality and morbidity from metabolic [23–25], infectious, gastrointestinal, and allergic diseases [7]. The potential health-protective mechanism may work through antibodies, nutrition, and gut microbiota. Although UK Biobank does not provide parent–offspring matched data, it collects data related to multiple early-life factors. With this dataset, researchers have revealed the associations between maternal smoking with various adult medical conditions, including cardiovascular diseases [21, 26], lung diseases [27, 28], mental conditions [12], and gastrointestinal conditions such as gallbladder diseases [29] and colorectal cancer [30]. In addition, the associations between breastfeeding and cardiovascular diseases [13, 26], mental conditions [12], asthma, and COVID-19 have also been investigated with these large-scale population-level cohort data. However, evidence is currently lacking for an association of the three perinatal early-life factors of interest with the risk of IBS in offspring.

The major strength of this study is the availability of several early-life exposures in a cohort with a large number of IBS cases. The collection of data was of high quality and reliability. We could also adjust for a wide range of potential confounders based on a Directed Acyclic Graph. It is worth noticing that being one of a multiple birth was considered a potential confounder in testing the effect of maternal smoking and breastfeeding, but the reverse was not true. The reason is the single-direction association of multiple births with maternal smoking and breastfeeding as assumed in the Directed Acyclic Graph.

A prior study proposed that breastfeeding could mitigate the adverse effects of maternal smoking on early brain development of the fetus [31]. Interactions between maternal smoking and breastfeeding have been noted in offspring’s psychological disorders [12] and hypertension [13]. Some researchers concluded that prolonged breastfeeding should be encouraged even if the mother smoked during gestation, as the breastfeeding duration modified the effect of smoking during pregnancy on the offspring’s risk of eczema [14]. We also investigated the joint effect of each pair of the three factors and the results indicated a generally additive effect without significant effect modification.

IBS is more common in females and younger populations, and whether sex and age modify the effect of certain risk factors is not fully understood. Furthermore, previous reports have identified offspring’s own smoking habits as a modifier of maternal smoking, which were revealed to influence the effect of maternal smoking on lung function and risk of chronic obstructive pulmonary disease [27] and cardiovascular events [21]. Therefore, we decided to test the effect modification by sex and age in all three factors and added offspring’s own smoking as a potential effect modifier in maternal smoking. The results suggested that the effect of maternal smoking around birth on IBS was modified by age only, while both age and sex of the offspring modified the effect of being breastfed as a baby.

We performed a series of sensitivity analyses, the rationale of which is explained below. First, although we identified several potential confounders, other covariates might act as mediators in the association between perinatal factors and IBS. For example, the offspring’s own lifestyle, such as smoking status, alcohol-drinking status, and BMI, could partly be a result of the mother’s smoking behaviour, while the offspring’s BMI could be also related to breastfeeding through nutritional mechanisms. Moreover, maternal smoking and breastfeeding could be interrelated, and these two could further be influenced by multiple birth. Therefore, we further adjusted one or more potential mediators. The effect of each factor slightly diminished, but maintained statistical significance after adjustment. Interestingly, being one of multiple birth had an independent significant effect on IBS and the underlying mechanism was unclear. Second, it is recognized that some organic conditions can be mistaken for IBS and there is growing appreciation that IBS can coexist with known but stable organic gastrointestinal diseases, such as quiescent IBD or coeliac disease [32]. Therefore, we further restricted participants to those without prevalent IBS, IBD, gastrointestinal cancer, coeliac disease, and other colitis, and the results remained similar. Third, more than half of the IBS cases were initially excluded because they were self-reported diagnoses at recruitment. Despite the concern about the quality of self-reported diagnoses, it is crucial to recognize that the exposures occurred at the early-life stage, making it unlikely that reverse causality could have significantly influenced the results. We again performed sensitivity analysis for the associations of the three exposures with both prevalent and incident IBS cases. The analysis further proved the robustness of the results from the main analysis.

This study has several limitations. Firstly, the UK Biobank cohort, in itself, has certain limitations. Over 30% of the participants were excluded because of unknown data on any exposure and the results might be biased if the missing data were not random. However, we observed that there was little difference in the demographical and lifestyle characteristics between the included and excluded individuals, although the *P*-values of the chi-squared tests were rather small due to the large sample size. Also, a comparable incidence of IBS between them provides further evidence that our exclusion criteria did not substantially affect the robustness of the conclusions. Lack of data on the duration and extent of maternal smoking and the duration of breastfeeding in UK Biobank limited detailed categorization of the exposures. Additionally, the impact of paternal smoking on offspring health is well documented [33, 34]; however, there are no available data on this field in the UK Biobank database and we cannot completely assess offspring’s early-life exposure to tobacco. We adjusted several socioeconomic and lifestyle-related covariates in our primary analysis, such as education level, Townsend Deprivation Index, smoking status, and drinking status, which are correlated with paternal smoking behaviour and may have partially adjusted for its potential effects to some extent. It is essential for future studies to directly assess the impact of paternal smoking on the risk of IBS in offspring. Although there was a data field on IBS family history, which was based on the digestive health questionnaire in part of the cohort, it was not included in the analysis due to nearly 65% missing data. Secondly, most of the participants were 40–60 years old when queried about early-life factors and such a long time inevitably leads to recall bias. However, three early-life factors in this study are not well-established risk factors for IBS and UK Biobank is not an IBS-specific cohort, which could to some extent mitigate the potential for recall bias. Thirdly, the diagnosis of IBS was based on International Classification of Disease codes instead of the standardized Rome IV diagnostic criteria, which may have introduced some human errors. Fourthly, UK Biobank primarily comprises middle-aged to elderly participants, which prevents us from studying the associations of these factors with IBS in younger populations. Although Sensitivity Analysis 3 included self-reported IBS before recruitment, there was no available information on the time of diagnosis. The predominantly White composition of UK Biobank participants also restricts generalizability to other ethnic groups.

## Conclusions

In summary, based on a large cohort study, our investigation revealed that individuals who were exposed to maternal smoking around birth and were one of a multiple birth exhibited an increased risk of offspring IBS in adulthood, while being breastfed as a baby acted as a protective effect against IBS. Future efforts are warranted to validate these findings.

## Supplementary Material

goaf042_Supplementary_Data
