# Supplementary material for: Association of maternal smoking, breastfeeding, and multiple birth with irritable bowel syndrome in older adults: a UK Biobank cohort study
Source: Gastroenterol Rep (Oxf). 2025 Jun 11;13:goaf042. doi: 10.1093/gastro/goaf042 (PMC12158159; doi:10.1093/gastro/goaf042)
Supplement: goaf042_Supplementary_Data [file goaf042_supplementary_data.zip › Supplementary material 20250427 final.docx]

**Supplementary Materials**

**Supplement Figure 1.** Directed acyclic graph. IBS, irritable bowel syndrome; BMI, body mass index.

**Supplementary Table 1.** Main characteristics of participants with and without complete data on three early life factors

| Characteristics | Participants with complete data on early life factors  (*n* = 334,586) | Participants with missing or unknown data on early life factors  (*n* = 156,293) | *P*-value |
| --- | --- | --- | --- |
| Age at recruitment, mean ± SD, years | 55.74 ± 8.14 | 58.25 ± 7.73 | 0.122 |
| Sex, *n* (%) |  |  | <0.001 |
| Male | 143,107 (42.8) | 83,053 (53.1) |  |
| Female | 191,479 (57.2) | 73,240 (46.9) |  |
| Ethnicity, *n* (%) |  |  | <0.001 |
| White | 297,320 (88.9) | 141,976 (90.8) |  |
| Non-white | 36,315 (10.8) | 13,453 (8.6) |  |
| Unknown | 951 (0.3) | 864 (0.6) |  |
| Townsend deprivation index, *n* (%) |  |  | <0.001 |
| Quartile 1 | 84,950 (25.39) | 37,827 (24.2) |  |
| Quartile 2 | 83,502 (24.96) | 38,974 (24.9) |  |
| Quartile 3 | 83,907 (25.08) | 38,905 (24.9) |  |
| Quartile 4 | 81,801 (24.45) | 40,395 (25.8) |  |
| Unknown | 426 (0.1) | 192 (0.1) |  |
| Education level, *n* (%) |  |  | <0.001 |
| Non-university | 240,600 (71.9) | 102,972 (65.9) |  |
| University | 41,731 (12.5) | 14,725 (9.4) |  |
| Unknown | 52,255 (15.6) | 38,596 (24.7) |  |
| Smoking status, *n* (%) |  |  | <0.001 |
| Never | 188,733 (56.4) | 78,411 (50.2) |  |
| Previous | 111,302 (33.3) | 57,750 (36.9) |  |
| Current | 33,511 (10.0) | 18,275 (11.7) |  |
| Unknown | 1,040 (0.3) | 1,857 (1.2) |  |
| Alcohol drinking, *n* (%) |  |  | <0.001 |
| Never | 15,843 (4.7) | 5,966 (3.8) |  |
| Previous | 11,460 (3.4) | 6,001 (3.8) |  |
| Current | 306,947 (91.7) | 143,034 (91.5) |  |
| Unknown | 336 (0.1) | 1,292 (0.8) |  |
| BMI, *n* (%) |  |  | <0.001 |
| Underweight | 1,742 (0.5) | 775 (0.5) |  |
| Normal | 111,865 (33.4) | 46,496 (29.7) |  |
| Overweight | 139,541 (41.7) | 68,037 (43.5) |  |
| Obese | 79,673 (23.8) | 39,672 (25.4) |  |
| Unknown | 1,765 (0.5) | 1,313 (0.8) |  |
| Incident IBS, *n* (%) |  |  | 0.185 |
| Yes | 7,254 (2.2) | 3,482 (2.2) |  |
| No | 327,332 (97.8) | 152,811 (97.8) |  |

BMI, body mass index; IBS, irritable bowel syndrome; SD, standard deviation.

**Supplementary Table 2.** Detailed description of covariates used in the study and related data field in the UK Biobank.

| Variable | Levels | Description | Related data field |
| --- | --- | --- | --- |
| Age | Continuous | Obtained from registry at recruitment | Field 21022 |
| Sex | Male/female | Obtained from registry at recruitment | Field 31 |
| Ethnicity | White/non-White/unknown | White includes British, Irish and any other with background; self-reported based on touchscreen questions at time of recruitment | Field 21000 |
| Education level | University/non-university/unknown | Obtained a college or university degree; self-reported based on touchscreen questions at time of recruitment | Field 6138 |
| Townsend Deprivation Index | Quartile1–Quartile 4 | Classified based on quartiles, a higher value indicates a lower socioeconomic status. Quartile 4 is highest. | Field 22189 |
| Smoking status | Never/previous/current/unknown | Self-reported based on touchscreen questions at time of image assessment visit | Field 20116, instance 2 |
| Alcohol drinking status | Never/previous/current/unknown | Self-reported based on touchscreen questions at time of image assessment visit | Field 20117, Instance 2 |
| Body mass index | underweight: <18.5 Kg/m² / normal: ≥18.5 and <25 Kg/m² / overweight: ≥25 and <30 Kg/m² / obesity: ≥30 Kg/m² / unknown | Constructed from height and weight measurements taken during the initial assessment center visit | Field 21001 |

**Supplementary Table 3.** Sensitivity analyses 1: additionally adjusted for potential mediators.

| Category of exposure | Adjusted | |
| --- | --- | --- |
|  | HR (95%CI) | *P*-value |
| Maternal smoking around birth |  |  |
| Further adjusted for breastfed as a baby | 1.21 (1.15, 1.27) | <0.001 |
| Further adjusted for own smoking | 1.21 (1.15, 1.27) | <0.001 |
| Further adjusted for own smoking, alcohol drinking and BMI | 1.20 (1.14, 1.26) | <0.001 |
| Further adjusted for all potential mediators | 1.19 (1.14, 1.26) | <0.001 |
| Breastfed as a baby |  |  |
| Further adjusted for maternal smoking | 0.94 (0.89, 0.99) | 0.016 |
| Further adjusted for own BMI | 0.92 (0.87, 0.97) | 0.002 |
| Further adjusted for both potential mediators | 0.94 (0.89, 0.99) | 0.018 |
| Part of a multiple birth |  |  |
| Further adjusted for maternal smoking | 1.22 (1.06, 1.40) | 0.007 |
| Further adjusted for breastfed as a baby | 1.20 (1.04, 1.38) | 0.012 |
| Further adjusted for both potential mediators | 1.20 (1.04, 1.38) | 0.011 |

BMI, body mass index; HR, hazard ratio; CI, confidence interval.

**Supplementary Table 4.** Sensitivity analysis 2: excluding participants with prevalent inflammatory bowel disease, gastrointestinal cancer, coeliac disease and other colitis.

| Category of exposure | No. of outcomes | No. of subjects | Unadjusted | |  | Adjusted | |
| --- | --- | --- | --- | --- | --- | --- | --- |
|  |  |  | HR (95%CI) | *P*-value |  | HR (95%CI) | *P*-value |
| Maternal smoking around birth^a^ |  |  |  |  |  |  |  |
| No | 4,647 | 235,797 | Reference | - |  | Reference | - |
| Yes | 2,249 | 91,939 | 1.30 (1.23, 1.36) | <0.001 |  | 1.22 (1.16, 1.29) | <0.001 |
| Breastfed as a baby^a^ |  |  |  |  |  |  |  |
| No | 2,062 | 89,000 | Reference | - |  | Reference | - |
| Yes | 4,834 | 238,736 | 0.71 (0.67, 0.75) | <0.001 |  | 0.91 (0.87, 0.96) | <0.001 |
| Part of a multiple birth^b^ |  |  |  |  |  |  |  |
| Singleton | 6,730 | 320,341 | Reference | - |  | Reference | - |
| Multiple | 193 | 7,395 | 1.26 (1.09, 1.45) | 0.002 |  | 1.23 (1.07, 1.42) | 0.004 |

^a^Adjusted for sex, age, ethnicity, education, Townsend deprivation index and part of a multiple birth.

^b^Adjusted for sex, age, and ethnicity.

HR, hazard ratio; CI, confidence interval.

**Supplementary Table 5.** Sensitivity analysis 3: including prevalent irritable bowel syndrome cases analyzed with logistic regression models.

| Category of exposure | No. of outcomes | No. of subjects | Unadjusted | | Adjusted | |
| --- | --- | --- | --- | --- | --- | --- |
|  |  |  | OR (95%CI) | *P*-value | OR (95%CI) | *P*-value |
| Maternal smoking around birth^a^ |  |  |  |  |  |  |
| No | 6,453 | 246,111 | Reference | - | Reference | - |
| Yes | 3,150 | 96,317 | 1.18 (1.15, 1.23) | <0.001 | 1.17 (1.13, 1.21) | <0.001 |
| Breastfed as a baby ^a^ |  |  |  |  |  |  |
| No | 2,909 | 93,267 | Reference | - | Reference | - |
| Yes | 6,694 | 249,161 | 0.84 (0.81, 0.87) | <0.001 | 0.90 (0.86, 0.93) | <0.001 |
| Part of a multiple birth^b^ |  |  |  |  |  |  |
| Singleton | 9,344 | 334,690 | Reference | - | Reference | - |
| Multiple | 259 | 7,738 | 1.15 (1.03, 1.27) | 0.010 | 1.13 (1.02, 1.25) | 0.021 |

^a^Adjusted for sex, age, ethnicity, education, Townsend deprivation index and part of a multiple birth.

^b^Adjusted for sex, age and ethnicity.

OR, odds ratio; CI, confidence interval.
