# Supplementary figures and images for: Association of maternal smoking, breastfeeding, and multiple birth with irritable bowel syndrome in older adults: a UK Biobank cohort study
Source: Gastroenterol Rep (Oxf). 2025 Jun 11;13:goaf042. doi: 10.1093/gastro/goaf042 (PMC12158159; doi:10.1093/gastro/goaf042)

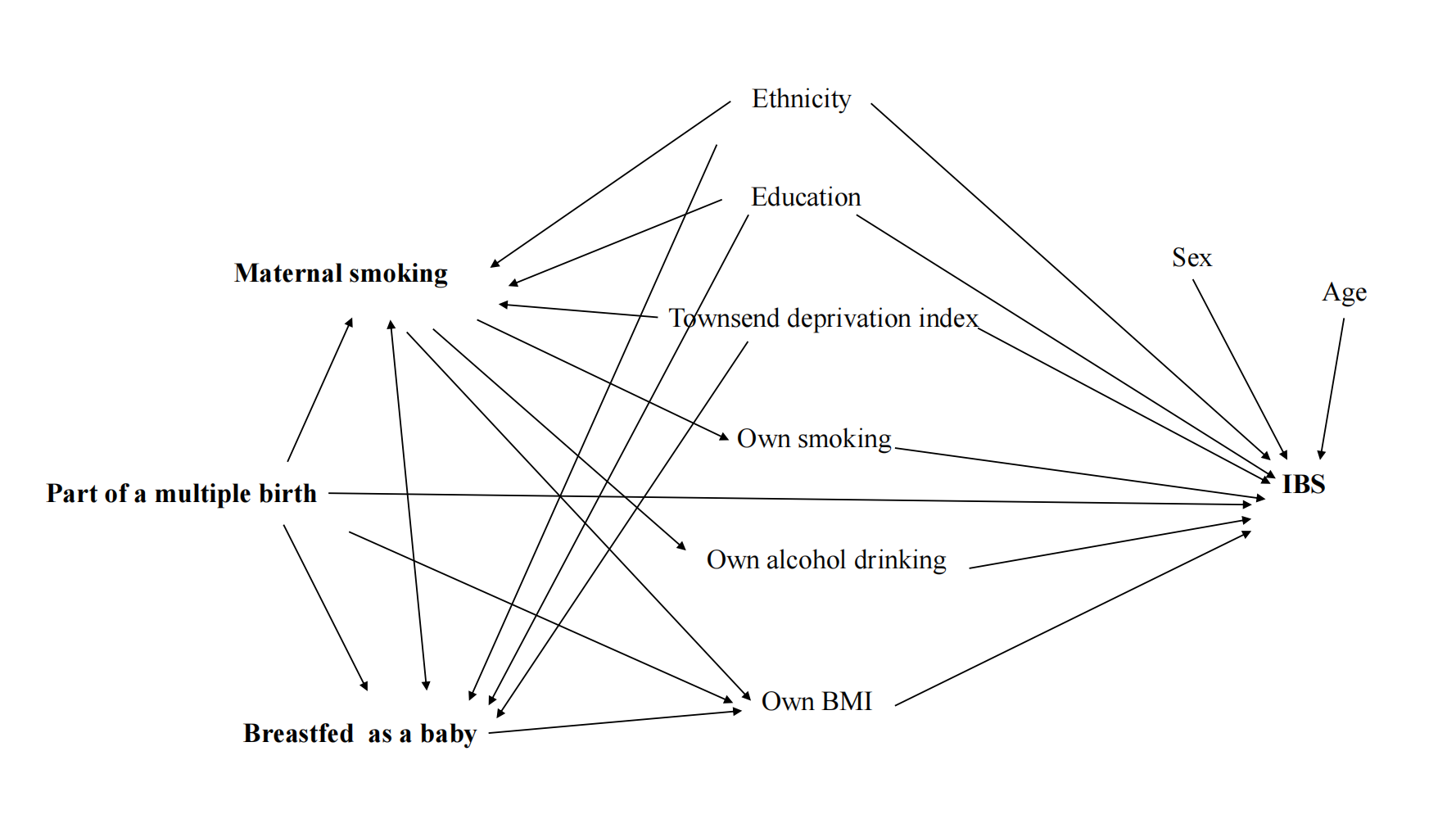

Supplement: goaf042_Supplementary_Data [file goaf042_supplementary_data.zip › Supplementary figure 1.tif]
